# Molecular dynamics simulations, molecular docking, and kinetics study of kaempferol interaction on Jack bean urease: Comparison of extended solvation model

**DOI:** 10.1002/fsn3.2956

**Published:** 2022-07-02

**Authors:** Leila Zolghadr, Gholamreza Rezaei Behbehani, Babak PakBin, Seied Ali Hosseini, Adeleh Divsalar, Nematollah Gheibi

**Affiliations:** ^1^ Department of Chemistry Faculty of Science Imam Khomeini International University Qazvin Iran; ^2^ Department of Food Hygiene and Quality of Control Faculty of Veterinary Medicine University of Tehran Tehran Iran; ^3^ Medical Microbiology Research Center University of Medical sciences Qazvin Iran; ^4^ Electrical Engineering Department Faculty of Engineering Imam Khomeini International University Qazvin Iran; ^5^ Department of Cell and Molecular Sciences Kharazmi University Tehran Iran; ^6^ Cellular and Molecular Research Center, Research Institute for Prevention of Non‐Communicable Diseases Qazvin University of Medical Sciences Qazvin Iran

**Keywords:** Jack bean urease enzyme, kaempferol, MD simulations, MM‐PBSA, molecular docking

## Abstract

Since the urease enzyme creates gastric cancer, peptic ulcer, hepatic coma, and urinary stones in millions of people worldwide, it is essential to find strong inhibitors to help patients. Natural products are well known for their beneficial effects on health and efforts are being made to isolate the ingredients, the so‐called flavonoids. Flavonoids are now considered as an indispensable component in a variety of nutraceutical, pharmaceutical, and cosmetic applications. Kaempferol (KPF) is an antioxidant found in many fruits and vegetables. Many reports have explained the significant effects of dietary KPF in reducing the risk of chronic diseases such as cancer, ischemia, stroke, and Parkinson’s. The current study aimed at investigating the inhibitory impact of KPF on Jack bean urease (JBU) using molecular dynamics (MD) simulations and molecular mechanics Poisson–Boltzmann surface area (MM‐PBSA) calculations to confirm the results obtained from isothermal titration calorimetry (ITC), extended solvation model, and docking software. In addition, UV–VIS spectrophotometry was used to study the kinetics of urease inhibition. Calorimetric and spectrophotometric determinations of the kinetic parameters of this inhibition indicate the occurrence of a reversible and noncompetitive mode. Also, the docking and MD results indicated that the urease had well adapted to the kaempferol in the binding pocket, thereby forming a stable complex. Kaempferol displayed low binding energy during MMPBSA calculations. The inhibitory potential of kaempferol was confirmed by experimental and simulation data, but in vivo investigations are also recommended to validate our results.

## Graphical abstract

Investigate the inhibitory effects of Kaempfrol on Jack Bean Urease using Kinetic study, Molecular Docking and MD simulation and its comparison with the thermodynamic results obtioned from extended solvation model. Adapting the results of extended solvation model and simulating molecular dynamics and investigation of interactions and select the best inhibitor and drug can help researchers in the fields of medicine for the treatment of diseases.

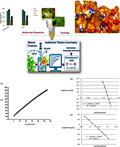

## 1 INTRODUCTION

Flavonols are flavonoids with a ketone group. They are building blocks of proanthocyanins. The most studied flavonols are kaempferol, quercetin, myricetin, and fisetin with specific clinical characteristics such as antiallergic, anti‐inflammatory, antifungal, antiviral, antitumor, anti‐*H. pylori*, and antibacterial properties(5–7) have recently attracted greater attention for treating cancer (Harris et al., 2016; Qiu et al., 2018). Kaempferol derivatives have also depicted antibacterial activity (10) and considerable inhibitory effects against the growth of *H. pylori* (11). Kaempferol (3,4′,5,7‐tetrahydroxy flavone), a yellow crystalline solid with a melting point of 276–278 °C (529–532 °F), is a natural flavonol, a kind of flavonoid, that is found in a specific variety of plants and plant‐derived foods such as kale, beans, tea, spinach, and broccoli (Holland et al., 2020; Sen et al., 2016). One of the most well‐known properties of Kaempferol is its anti‐inflammatory and anticancer properties. Urease (urea amidohydrolase; E.C. 3.5.1.5) is a nickel‐containing enzyme that catalyzes the hydrolysis of urea to the formation of ammonia and carbon dioxide (Mobley et al., 1995). The ammonia that is produced there does serious damage to gastric epithelium through its interactions with the immune system in humans while also creating severe metabolic disorders (Preininger & Wolfbeis, 1996; Suzuki et al., 1992). Also, urease creates several pathogenic states in both humans and animals, such as gastric cancer, stone formation in kidneys, urinary and GIT infections, pyelonephritis, ammonia encephalopathy, catheter encrustation, hepatic coma (Aidoo et al., 2013; Ciurli et al., 1999; Jang et al., 2008; Mobley et al., 1995). The high activity of urease in agriculture creates major environmental and economic problems by releasing abnormally large amounts of ammonia into the atmosphere during urea fertilization. Furthermore, the high activity damages plant essentially by robbing plants of their essential nutrients and creating ammonia toxicity thereby increasing the pH of the soil (Bremner, 1995). Urease inhibitors making up several compounds, e.g., hydroxamic acid and its derivatives, were used in agriculture and medicine (Muri et al., 2004). Imidazole derivatives (Kühler et al., 1995), dicoumarols (Khan, Iqbal, Lodhi, Maharvi, Shahnaz Perveen, et al., 2004), biscoumarin (Khan, Iqbal, Lodhi, Maharvi, Shahnaz Perveen, et al., 2004), a‐hydroxy ketones (Tanaka et al., 2004), polyphenols (Xiao et al., 2007), and several oregano bismuth compounds (Murafuji et al., 2006), have also been investigated in terms of their effects on urease inhibition. Bioinformatics plays an essential role in drug development. The discovery of herbal drug candidates requires a strong assessment of pharmacological quality, including absorption, distribution, metabolism, excretion, and toxicity (Ferreira et al., 2015). The inhibition mechanism has not been fully understood despite urease being the first crystallized enzyme. The logical design of urease inhibitors is strongly enforced by the knowledge of crystal structures of this enzyme. From the published crystal structures, it can be understood that the presence of nickel‐complexing moiety alongside properly placed, network of hydrogen‐bond donors and acceptors attached to flexible scaffold is effective in inhibiting this enzyme. Several an in silico investigations were performed with the aim of finding potent inhibitor molecules that could strongly bind to the active site of urease; such as (PDB4H9M, Kaempferol 3 ‐O‐(6”‐O‐trans‐coumaryl) glucoside 7‐O‐(6″’‐O‐trans‐coumaryl) glucoside, Kaempferol‐3‐O‐(6”‐O‐trans‐coumaryl) glucoside (Tiliroside), (PDB 4GY7), carbonic anhydrase‐II from *Iris* species), (PDB 4H9M, Luteolin) or (PDB 4CEX, fluoride) (Benini et al., 2014; Eftekhari et al., 2021; Mazinani et al., 2020; Saleem et al., 2019). Also, an in silico evaluation of bioactive molecules of tea such as kaempferol indicated that it can be a strong inhibitor of protein‐15 SARS‐COV‐2 (Sharma et al., 2021).

In this study, we will challenge our previous thermodynamic results of kaempferol binding to urease, performed with isothermal titration calorimetry (ITC) and an extended solvation model (Zolghadr & Behbehani, 2019). A previous in vitro study reported that kaempferol was a good inhibitor; therefore, the power of molecular dynamics and molecular docking was used for confirmation. We were using the GROMACS 4.5.4 package and Auto Dock 4 (version 1.5.6) software and inhibition kinetics investigation through molecular dynamics evaluation. After MD simulation studies, the MM‐PBSA approach was applied to interpret the free binding energy.

## 2 MATERIALS AND METHODS

Kaempferol and purified Jack bean urease were purchased from Sigma.

### 2.1 Kinetic studies

Calorimetric experiments were carried out with a high‐sensitivity ITC (ITC: isothermal titration calorimetry) micro‐calorimeter (Thermal Activity Monitor 2277, Thermometric, Sweden). The Hamilton syringe was used to inject kaempferol solution (8000 μmol/L) into the vessel containing 5 μmol/L enzymes, and 30 injections were performed consecutively. In the next step, the heat of each injection was calculated by the “Thermometric Digitam3” software program. In addition, the heat of the biomolecules ligands’ interactions (q) in the aqueous solvent system could be precisely calculated through the following Equation:
(1)

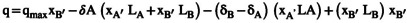




The inhibitory effect of kaempferol on Jack bean urease was investigated in a study to examine the kinetic studies, inhibitory potential, and mechanism of inhibition in phosphate buffer and 1 mM EDTA at pH 6.8. Lineweaver–Burk plots were constructed from kinetic data to determine the mechanism of enzyme inhibition by varying the concentrations of substrate urea in the presence of different concentrations of kaempferol compound (0, 0.5, 1, 2.5, and 5 μM). Inhibition constant (*K*_i_) was determined, different concentrations of inhibitor were obtained from the Lineweaver–Burke plot, and all experiments were conducted in triplicate. The IC50 values of inhibitors were calculated. Inhibition was found noncompetitive.

### 2.2 Protein–ligand docking process

To get insight into possible interaction modes between active sites of Jack bean urease, the molecular docking study was performed using AutoDock4 software. We obtained geometries of kaempferol from PubChem (CID: 5280863), while the 3D structure of receptor urease (jack bean) was obtained from protein data bank (PDB) with PDB4H9M (http://www.rcsb.org). The molecules of water, acetohydroxamic acid, and 1,2‐ethanediol were cleaned from the enzyme structure, and hydrogens were added while calculating Gasteiger charges. The co‐crystalized ligand of the receptor with a grid box of center *x* = 19.067, *y* = −56.327, and *z* = −21.334 and size of *x* = 70 °A, *y* = 66 °A, and *z* = 64 °A identified grid center. Auto Dock was run several times (here 500 times) as previously described. Also, all connection states resulting from molecular docking were grouped in one cluster. The RMSD size was also considered for clustering 2^°^A. Kaempferol molecular docking to urease was examined through a commutable docking process. The ligands had enough flexibility and could interact with urease chains through Auto Dock (version 4.2). LIGPLOT+ version v.1.4.5 software, discovery studio visualizer version 4.0, PyMOL version 1.7.2, and Chimera were used to analyze predicted docked poses of kaempferol against urease enzyme.

Through Auto Dock, the lowest binding energy of kaempferol–urease complexes for docking conformation was determined and assumed as early conformations for MD simulations and MM‐PBSA calculations.

### 2.3 Molecular dynamics simulation and MM‐PBSA analysis

The molecular dynamics simulation process of the studied complex was carried out using GROMACS 2020.1 software suite (Hess et al., 2008). For the water solvent model, TIP3P and force field are also CHARMM (Jorgensen et al., 1983). To perform the simulation in the neutralization and minimization stages of the system, the (Steep = Steepest descent minimization) algorithm was used for integration into 50,000 steps. Then, the equilibration steps were performed with the help of canonical (NVT) and isothermal–isobaric (NPT) ensembles with the help of the Leap‐frog algorithm for integration into 500,000 steps (Hockney, 1970). The system configuration was saved every 0.2 ps. The restriction was also performed on all links. The primary sampling step was performed with the help of an NPT set for 30 ns. To fix the temperature of the system at 300 K, the Nose–Hoover thermostat constant was applied. The Parrinello–Rahman pressure coupling method was applied to preserve the system pressure at a fixed 1 bar. The structure of the rigid water model was constrained using SETTLE (Vermaas et al., 2016). The biding of bond length containing hydrogen atoms was performed using the LINCS algorithm (Hess et al., 2008). Also, to measure the electrostatic interactions, the particle mesh Ewald (SPME) method was applied with 1.0 nm short‐range electrostatic and van der Waals cut‐offs (Essmann et al., 1995). Finally, while taking time steps of 2 fs on equilibrated systems, 100 ns MD simulation for the complex of urease‐ kaempferol was done. The stored paths in the simulation are used to analyze the structural parameters of the studied complex. In the next step, six parameters including analysis of hydrogen bonds (H‐bonds) and surface accessible solvent area (SASA), the root mean square deviation (RMSD), root mean square fluctuation (RMSF), radius of gyration (*R*_g_), and dictionary of secondary structure of protein (DSSP) were studied.

### 2.4 MMPBSA calculations

The molecular mechanics Poisson–Boltzmann surface area (MMPBSA) approach has been widely applied as an efficient and reliable free energy simulation method to model molecular recognition, such as for protein–ligand binding interactions (Arba et al., 2017; Duan et al., 2016; Sharma et al., 2021). The gmmpbsa module of GROMACS was used for MMPBSA calculations on the simulated urease–kaempferol complex to obtain the final binding free energies and residue contribution energies of the complex (Kumari et al., 2014). Equations were used in this module for calculation of binding free energy (BFE) as follows:

$${\text{∆G}}_{\text{binding}}=\text{∆H}-T∆S.$$

$$\text{∆H}={\text{∆E}}_{\text{electrostatic}}+\text{∆E}\;{\mathrm{vd}}_{W}+{\text{∆G}}_{\text{polar}}+{\text{∆G}}_{\text{polar}}.$$

In these equations, ∆G _binding,_ ∆E _electrostatic,_ ∆E vd _W,_ ∆G _polar_, and ∆G _polar_ are the BFE, the electrostatic contribution, the Vander Waalse contribution, and polar and nonpolar solvation terms, respectively. The nonpolar solvation term is more commonly referred to as the SASA contribution (Kumar et al., 2021).

### 2.5 RESULTS AND DISCUSSION

Previously, it was demonstrated by Behbehani et al. that the heats of the biomolecules ligands interactions (q) in the aqueous solvent system could be precisely calculated through the following Equation (Behbehani et al., 2008; Rezaei Behbehani et al., 2011; Rezaei Behbehani, Saboury, Barzegar, et al., 2010):

In this research, we used this equation and isothermal titration calorimetry (ITC) to study the thermodynamics of kaempferol binding to urease. To further confirm our results, we investigate and describe inhibition using kinetics studies and mechanism of inhibition and molecular dynamics simulations, and MM‐PBSA calculations in the present study.

### 2.6 Investigation of **positive cooperativity of** kaempferol with urease

The inhibition of urease by kaempferol was investigated using isothermal titration calorimetry (ITC), a method that is becoming a major tool to aid enzyme inhibitor screening and design. As mentioned, we used Equation _1_ to calculate the heats of the JBU + KPF interactions. To investigate the type of ligand–enzyme cooperation, we needed to obtain the value of the parameter P. We used the following two equations:

$$X^{\prime }B=\frac{\mathrm{PXB}}{\mathrm{XA}+\mathrm{PXB}\;}\mathrm{XB}=\frac{\left[\text{kaempferol}\right]}{\left[\text{kaempferol}\right]\max}$$

**FIGURE 1:**
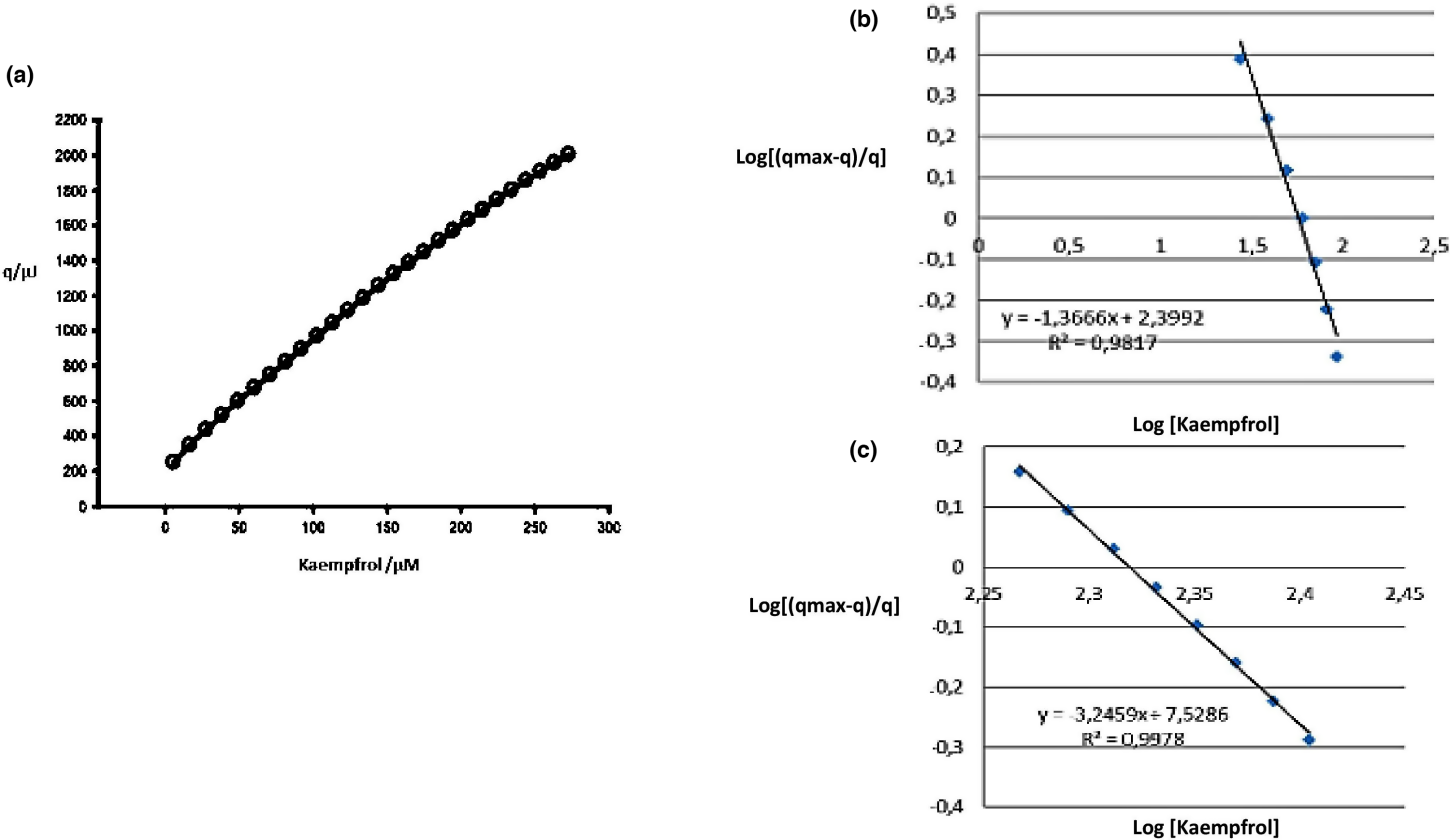
(a) The experimental heats (▵) at 300 K, for (JUB+ KPF). (b,c) The fitting of heat of JBU + KPF in low and high concentrations, respectively

The results of fitting the heat of urease and kaempferol interactions are shown in Figure (1a) and Table 1. The analysis results indicated that hydrophobic forces are the dominant interactions between urease and Kaempferol. The negative numerical values δ_A_ and δ_B_ and also their low numerical difference (−0.23 and − 0.17) show minor changes in the structure of urease and instability of the urease–kaempferol complex and specific interactions, respectively. On the other hand, *p* > 1 confirmed positive cooperativity of kaempferol with urease (Table 1). By changing the logarithm in Equation _1_, we can bring it closer to the Hill equation, and we continued our studies in the high and low concentration regions of kaempferol.

**TABLE 1:** Binding parameters for JBU+ Kaempferol interaction from Equation _1_

Parameters	*p*	Ka/Lmol^−1^	ΔH/KJmol^−1^	ΔG/K Jmol^−1^	TΔS K Jmol^−1^	δA	δB
	1.2	253,009	5.16	−31.03	36.19	−0.23	−0.17

The results of Hill equation fitting in the low and high concentration regions of Kaempferol are reported in Table 2 and Figure (1.b, c).

**TABLE 2:** Thermodynamic parameters for Kaempferol +JBU interaction from Hill Equation _1_

Regions	*n*	Ka/Lmol^−1^	ΔH/KJmol^−1^	ΔG/K Jmol^−1^	TΔS K Jmol^−1^
Low [kaempferol]	1.37	5.65 × 10^7^	4.52	−44.62	49.15
High [kaempferol]	3.25	2.46 × 10 ^8^	4.15	−48.31	52.83

As you can see in Table 2, the value of n in two regions is more significant than 1 (*n* > 1) and confirms this positive cooperation, which is the same result we obtained from Equation _1_.

### 2.7 Kaempferol noncompetitive inhibition of urease

**FIGURE 2:**
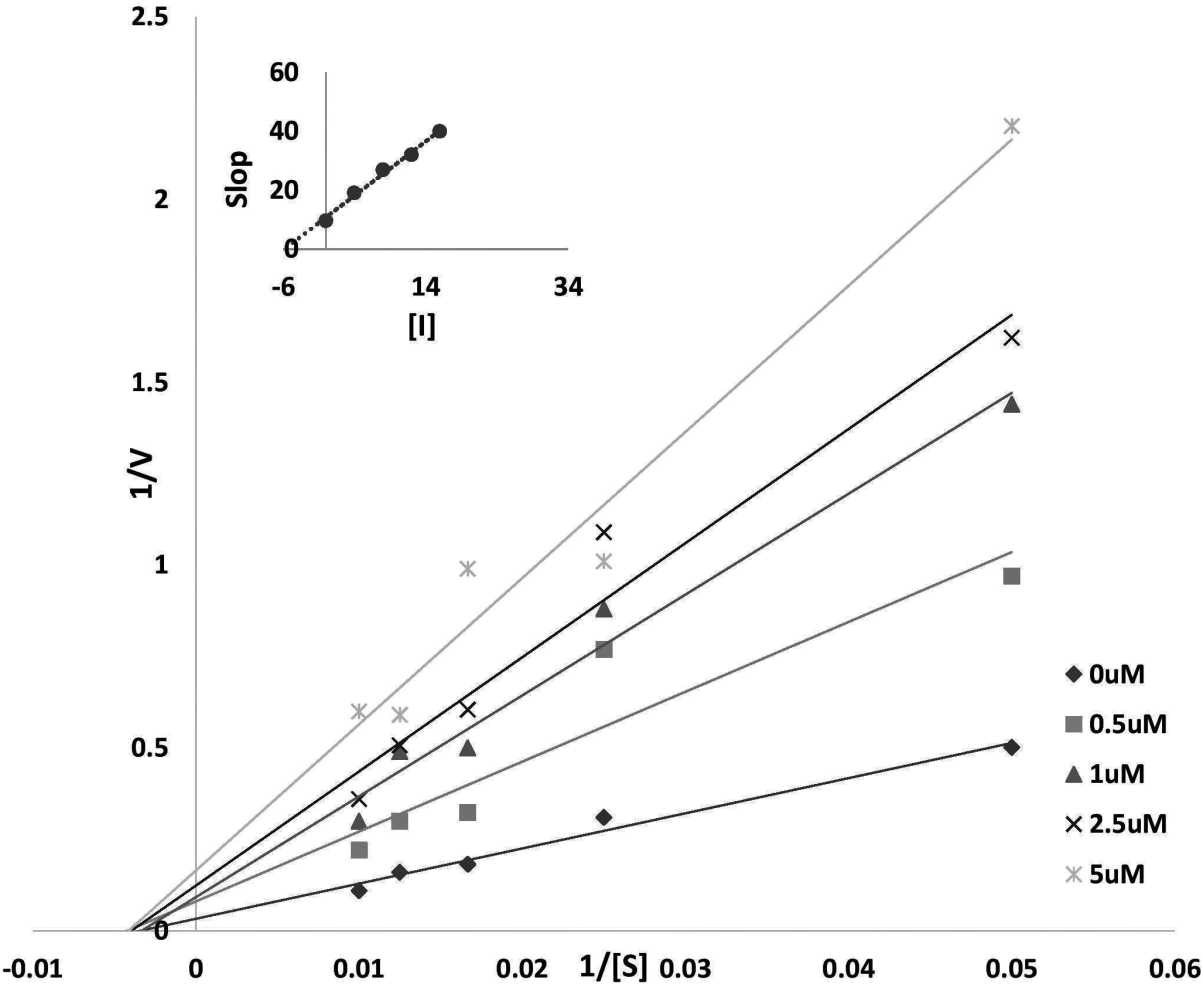
Lineweaver–Burk plot for kaempferol (0, 0.5, 1, 2.5, 5 μM)

The results of our kinetic studies showed that urease activity is dose dependent. The IC_50_ value was calculated as 6.96 μM; the results showed reversible inhibition since all straight lines passed through the origin. Lineweaver–Burk analysis was performed, and inhibition was evaluated. The change in Vmax showed that the inhibition was noncompetitive while Km was constant. The *K*_i_ value was 6.06 μM (Figure 2). The study of the kinetics of Morin (3, 5, 7, 2′, 4′‐pentahydroxyflavone), that is “a bioflavonoid” by Ritu Kataria et al., in 2019 had a similarity to result from the present study. It confirmed the noncompetitive inhibition (Kataria & Khatkar, 2019). It has been reported that copper ion and nickel ion as salt showed the IC_50_ value of 1.71 ± 0.56 μM and 8.01 ± 1.21 μM compared to acetohydroxamic acid (AHA, IC50 = 26.99 ± 1.43 μM); however, 2‐[4‐(4‐fluorophenyl) pi‐perazin‐1‐yl] acetic acid (HL) showed no urease inhibitory activities. Zhi‐Jian Chen et al. reported the urease inhibitory activity of HL as weaker than those of organic compounds synthesized by Sheng et al. (Chen et al., 2016; Sheng, Chen, et al., 2015). It appears that electronic configurations, ligand substituents, and the nature of the center of metal influenced the inhibitory activities of metal complexes (Chen et al., 2010; Sheng, Zhou, et al., 2015; You et al., 2010).

### 2.8 Molecular Docking (Interaction analysis)

**FIGURE 3:**
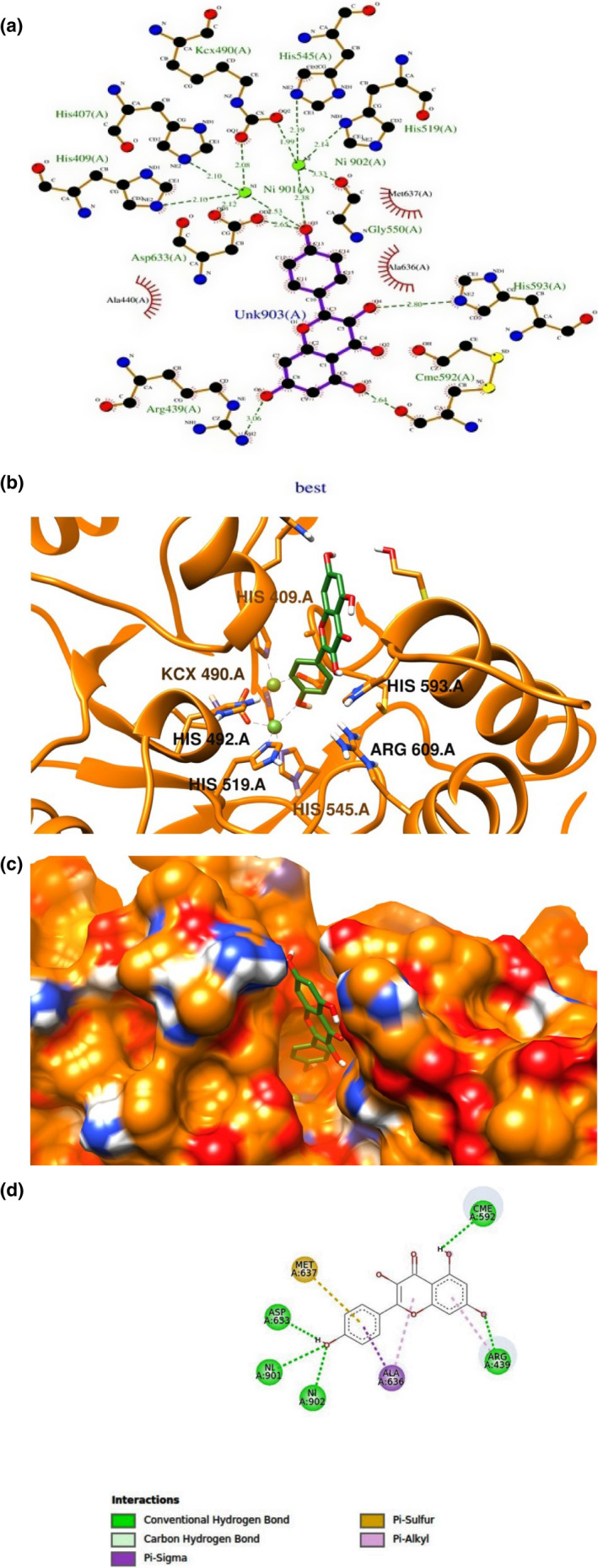
Binding interactions of kaempferol with the active binding site of urease PDBID 4H9M generated using Ligplot and discovery. (a, c) Show the 3D docking of kaempferol in a binding pocket (b). (d) Shows the two‐dimensional ligand–protein interactions. Legend inset represents the type of interaction between the ligand atom and the amino acid residues of the protein

No published reports have described the urease–kaempferol. Thus, this work aimed to gain further insight into the working molecular mechanism of the inhibitory effect of kaempferol on *Jack bean urease*. Initially, in the current study, the interaction between kaempferol and specific binding sites on urease through hydrogen bonding, metal/ion contact with Ni ions, and hydrophobic interactions was evaluated by conducting molecular docking. The critical location of kaempferol with urease has excellent importance since gaining insights into protein–ligand binding interactions can help us to understand the function and efficacy of kaempferol functioning as potential therapeutic agents. The AutoDock4 software has key features such as selective side‐chain residue flexibility, essential to the present study (Abreu et al., 2012). The main advantage of this option is providing a more pragmatic approach to the interaction of ligand–protein without any significant increase in computer processing time. The conformational modifications that occur within the receptor may impart major concepts and demonstrate that receptor flexibility is an important part of computational drug design that has been mentioned above (Mohan et al., 2005). Table S1 and Figure S1 summarize all details related to the docking study of kaempferol in the binding site of urease. Based on our results, *K*_i_ and binding energy were equal 17.92 μM and − 6.48 kcal/mol, also the amount of vdW energy between urease and ligand is about −7.79 kcal/mol, and in comparison, with electrostatic energy (−0.17 kcal/mol) plays a very important role in the complex. The computational molecular docking results showed that kaempferol interacts with two Ni_901‐902_ atoms by the phenyl rings and is surrounded by the amino acids His519, His492, Kcx490, Arg609, His593, and His409. Also, hydroxyl group in phenyl ring has established two hydrogen interactions with two Ni atoms. In addition, four hydrogen interactions with Arg439, Cme592, His593, and Asp633 amino acids with ligand were observed, while there are three hydrophobic contacts from the residues Ala 440, Ala 630, and Met 637 (Figure 3a–c). The literature supports our docking results due to the presence of such functionalities (Saeed, Channar, et al., 2017; Saeed, Mahesar, et al., 2017; Saeed, Rehman, et al., 2017). The interactions of kaempferol with urease were explored by analyzing its binding pattern. Kaempferol interacted with three of the critical residues of urease active site, namely Asp633, Cme 592, and Arg439, forming one conventional H bond with Asp633, one conventional H bond, and one carbon H bond with Cme592; one conventional H bond, one‐carbon H bond, and pi‐alkyl with Arg439; and Pi‐sulfur with Met‐637. Apart from these residues, it has also interacted with Ala636, forming one pi‐sigma bond and one pi‐alkyl bond (Figure 3d).

Recently, studies have been performed on the inhibition of kaempferol derivatives on Jack bean urease. In the year 2021, Eftekhari and et.al indicated that the hydroxyl groups of kaempferol moiety in the tiliroside were also engaged in the binding cavity through nickel ions (2.14 Å, 2.67 Å) hydrogen bonds with the oxygen atom of Asp633 and Cme592side chains (Eftekhari et al., 2021); they show that electron‐rich hydroxyl groups played vital roles in the urease inhibitory activity, for example, in *Kaempferol 3‐Oβ ‐Oneohesperidoside‐7‐O‐[2‐O‐(cis‐feruloyl)] β ‐D‐glucopyranoside* compound. The OH group of the neohesperidoside moiety mediated bidentate interactions with Ni ions, while the kaempferol part an inert which may be attributed to the space hindrance of some groups, resulting in moderate inhibition (Eftekhari et al., 2021). Our results showed that the hydroxyl groups of rings of kaempferol interacted with crucial residues and Ni ions. Moreover, eftekhari et al. showed *kaempferol‐3‐O‐(6”‐O‐trans‐coumaroyl) glucopyranoside 7‐O‐6′ coumaroyl glucopyranoside* inhibits urase with a free binding energy equal to −6.11 kcal/mol and *K*_i_ equal to 33.28 μM, the hydroxyl groups of rings no interacted with crucial residues and Ni ions (Eftekhari et al., 2021). In the present study, kaempferol with the lowest free binding energy −6.48 kcal/mol showed an appropriate docking score and suitable interactions in agreement with the biological activity, suggesting that kaempferol could be an efficient inhibitor in comparison to *kaempferol‐3‐O‐(6”‐O‐trans‐coumaroyl) glucopyranoside 7‐O‐6′ coumaroyl glucopyranoside* and *Kaempferol 3‐Oβ ‐Oneohesperidoside‐7‐O‐[2‐O‐(cis‐feruloyl)] β ‐D‐glucopyranoside* **(**free binding energy = −1.60 kcal/mol**).** To sum up, these synthetic compounds bearing bulky substitutions were understood to be weaker inhibitors than kaempferol. The OH group of the neohesperidoside moiety of these compounds mediated bidentate interactions with Ni ions, while the kaempferol part of these compounds showed an inert behavior which may be attributed to the spatial hindrance of some groups, resulting in weak inhibition. In another report, evaluation of different bioactive molecules of tea for inhibition potency SARS‐COV‐2, results molecular docking results, indicated that kaempferol could be considered as potential inhibitor of NSp15 (total binding energy: −12.9 kcal/mol) (Sharma et al., 2021). Weak intermolecular interactions such as hydrogen bonding and hydrophobic interactions are key players in stabilizing energetically favored ligands, in an open conformational environment of protein structures (Patil et al., 2010). Our results indicate that both interactions stabilize the kaempferol at the active site, and help alter binding affinity.

### 2.9 MD simulations and MM‐PBSA analysis

**FIGURE 4:**
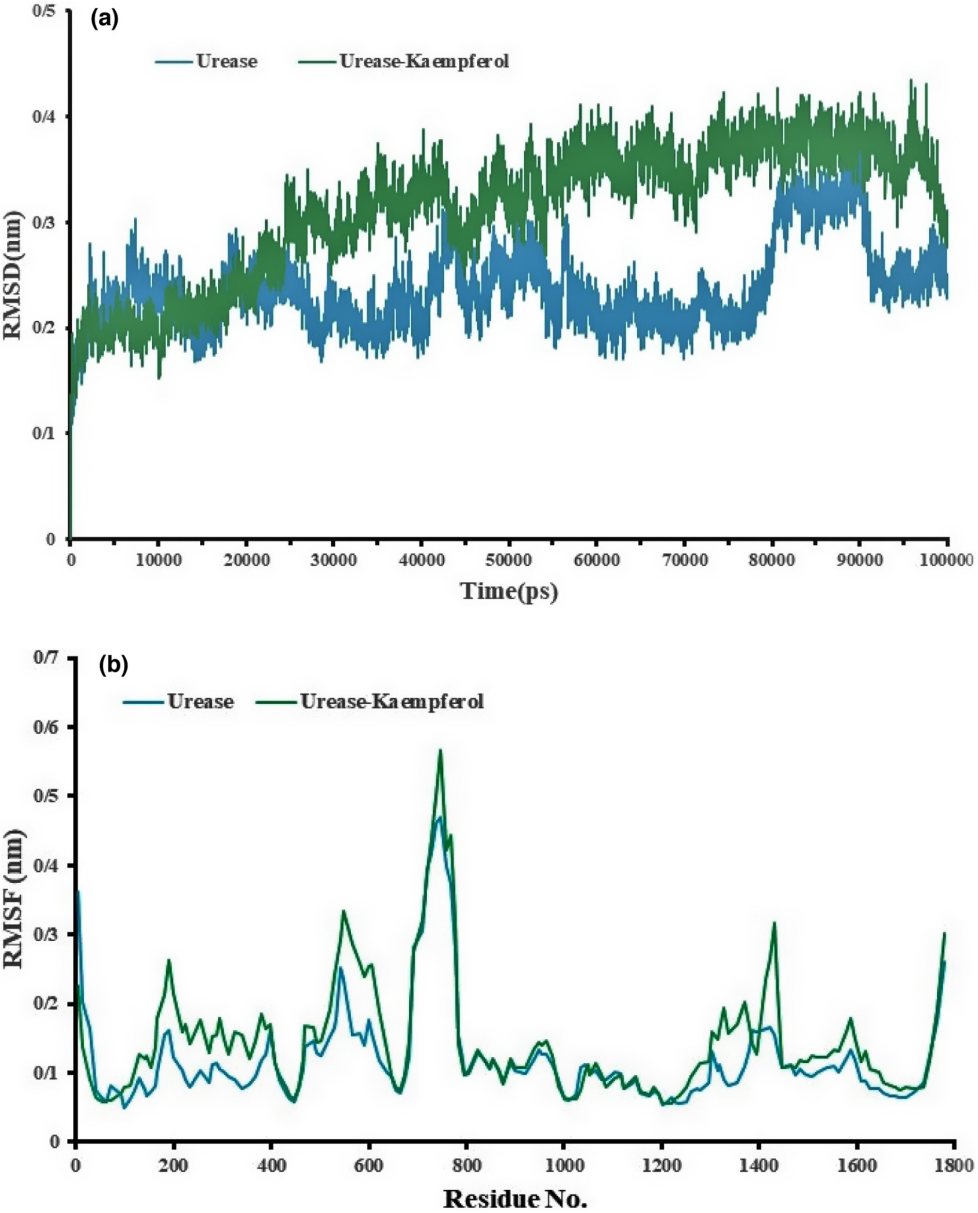
(a): RMSD as a function of simulation time and (b): RMSF as a function of amino acid residues

MD simulations are important to closely examine the stability, conformational changes, internal motions, etc. of protein–ligand complexes, which have been shown to be effective in mutational analysis and inhibitor designing (Gupta et al., 2021; Shin et al., 2020). The MD simulations of free urease and urease–kaempferol complex were performed, and the results were compared to measure the structural changes caused by ligand binding. RMSD is deemed a crucial structural and dynamical parameter to evaluate the conformational stability despite investigating the quality, equilibration, and convergence of an MD run (Gupta et al., 2021). A more considerable RMSD value demonstrates the lower strength of a protein complex and contrariwise. On the other hand, higher fluctuations indicate low stability. Highly deviated RMSD graphs can also imply major conformational transitions occurring in the protein to obtain stable conformation with the ligand. In this study, the RMSD of the kaempferol–urease complex and urease free concerning the Cα atom was calculated versus time, Figure 4a. In the case of the urease, the average RMSD is about 0.3023 nm, with some fluctuations at around 8 ns and 7.3 ns (Figure 4a). Whereas in the kaempferol–urease complex, the average RMSD is about 0.45 nm. Strong changes have been observed at 50–80 ns. The system is not in equilibrium when the RMSD of urease is increasing or decreasing after MD simulation. Thus, the duration of the MD simulation may be insufficient for robust analysis (Sharaf et al., 2022). The higher fluctuation of the RMSD of urease and higher average value in complex indicate that the urease–kaempferol complex structure is changed, thus suggesting bonding between the two. The residues of the protein play a vital role in achieving a stable conformation for a protein–ligand complex, which can be gauged by using the RMSF as a parameter. RMSF graphs showing higher levels of RMSF imply increased flexibility which in turn indicates their increased potential to interact with the ligand molecule. Similarly, lower RMSF fluctuations imply lesser flexibility, hence diminished interaction potential. Additionally, regarding the backbone atoms of each amino acid residue of urease in the complex and free urease presented in Figure 3a, b, RMSF proves to be a valuable parameter to estimate residue flexibility during dynamics. Regarding urease without ligand, the average RMSF is about 0.49 nm. Aside from the starting and terminal residues, some higher spikes at the residues 189–760 could be seen as presented in the plot of Figure 4b. Similarly, concerning the urease–kaempferol complex, with the average RMSF of 0.56, the spikes mostly emerge in the RMSF plot in different previous locations (Figure 4b). As we know Jack bean urease consists of four domains having different amino acids length: Domain 1: amino acids (1–124), Domain 2: amino acids (136–147, 272–401, and 701–753), Domain 3: amino acids (150–262), and Domain 4: amino acids (402–689 and 724–828). Domain 4 is an active region of the target protein with embedded two Ni atoms (Saeed, Mahesar, et al., 2017). As you can see in Figure 4b, the highest average fluctuations are observed in the active site areas (His409, His 519, Glys550, Asp633, Arg439, Cme 592, and Met 737). This result was acceptable given that we focused on the dynamic behavior of the active site of urease. Moreover, two significant peak fluctuations were also observed by the residues other than the active site residues, indicating their increased interaction potential implying that the kaempferol able to adapt well in the binding pocket of the urease.

**FIGURE 5:**
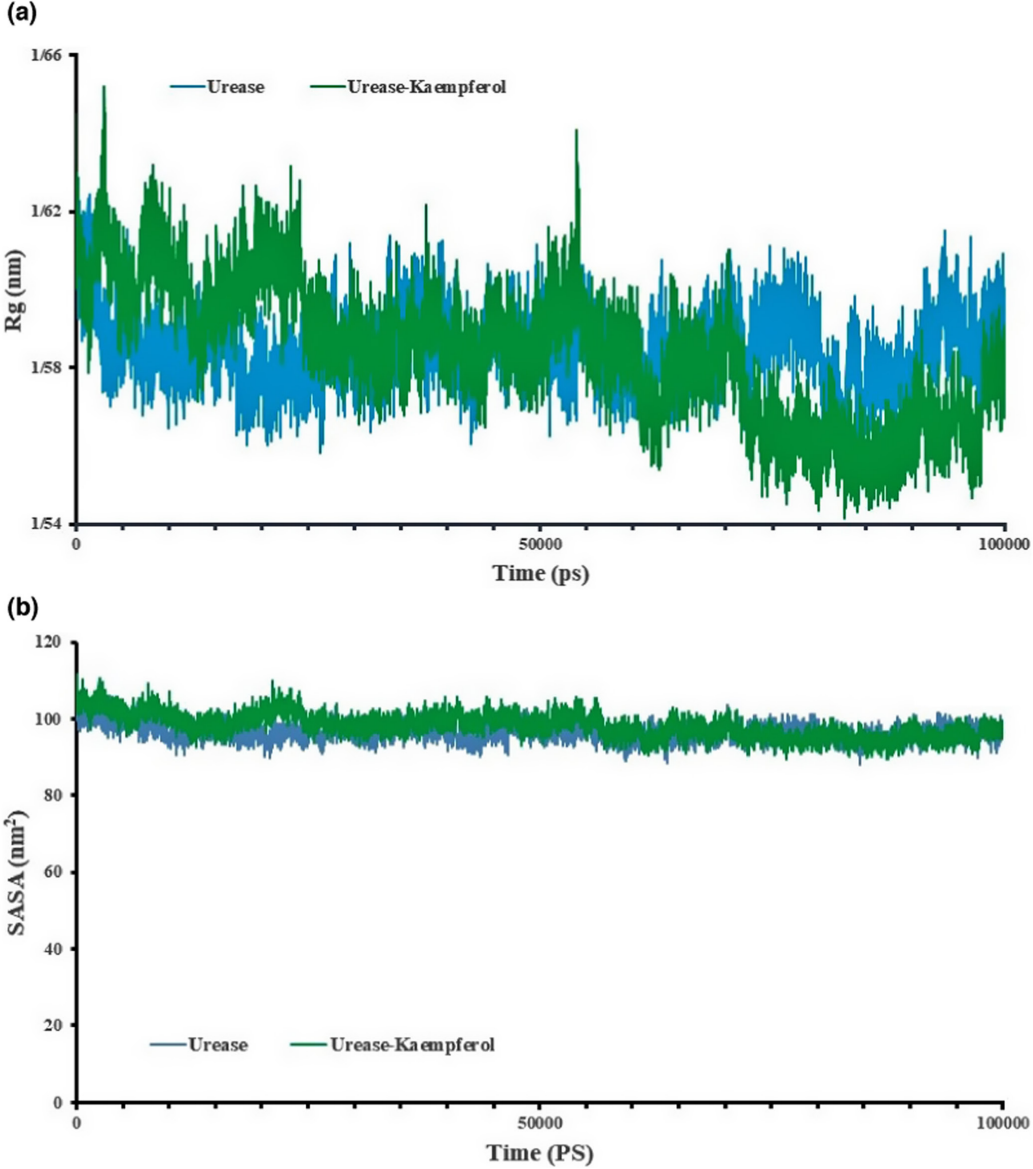
(a): *R*_g_ as a function of simulation time and (b): SASA as a function of simulation time

**FIGURE 6:**
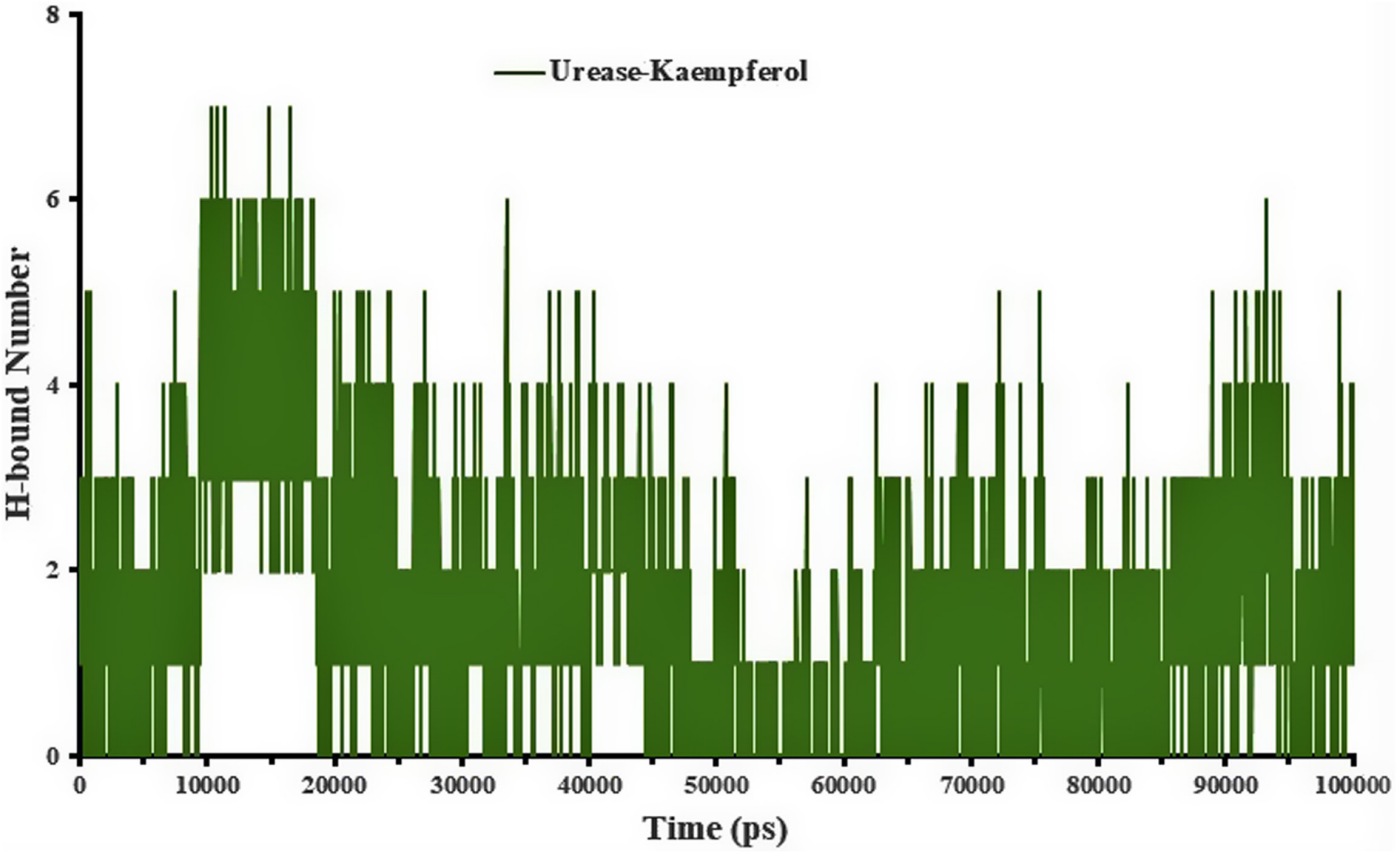
The average number of hydrogen bonds as a function of simulation time for urease–kaempferol complex

The radius of gyration (*R*_g_) determines the protein compaction level. According to its definition, it is considered as the mass‐weighted root‐mean‐square distance of a set of atoms from their common center of the mass. Hence, the evolution of the overall protein dimension during dynamics is represented by the trajectory analysis of the radius of gyration. Since it is not easy to determine the relative distance of each atom to the center of mass of the protein, we use the radius of gyration (*R*_g_) factor to investigate the folding or unfolding of the protein. As shown in Figure 5a, the *R*_g_ of urease is greater than *R*_g_ of the urease–kaempferol complex at the end of the simulation time when the system reaches equilibrium. The urease–ligand structure saw four significant ascents of 53 ns. In less than 19 ns, the *R*_g_ of complexation increases, related to hydrophobic interactions. The *R*_g_ increases from 1.58 to 1.65, making the system more open. The change in system state from the fold to open indicates the system’s instability. After 50 ns, little change is seen, which means the system is in equilibrium. Totally, concerning urease–ligand, the average value of *R*_g_ is 1.6502 nm, and the ascent is significant with a size of about 3.2 ns (Figure 5a). However, concerning the urease, the average value of *R*_g_ is about 1.58 nm, and different decays occur in the *R*_g_ plot, as presented in Figure 5a. In general, MD results showed that urease in the complex is more unstable than urease in the free state, and these fluctuations are evident in the protein complex. The surface accessible solvent area (SASA) is of particular importance in the interaction of the protein with water, other ligands, other proteins, or the structure of the protein itself. This parameter is often calculated to evaluate the structure of the protein. In this study, the SASA is calculated in 10,000 ps. This is consistent with the surface accessible solvent area (SASA) data in Figure 5b. As shown in Figure 5b, the accessible surface of hydrophobic amino acids in the kaempferol–urease complex is higher. Hydrogen bonds formed between ligand and protein are responsible for maintaining a compact and a properly oriented structure, where the flexibility of the protein’s residues also comes into play as they are the ones that will be forming bonds with the ligand molecules. The number of H bonds formed by the urease with the kaempferol was calculated and depicted on a graph, as shown in Figure 6. Kaempferol formed a maximum of ~7 H bonds with the urease and a minimum of 0 H bonds, whereas the average number of H bonds decreased as the simulation progressed.

**FIGURE 7:**
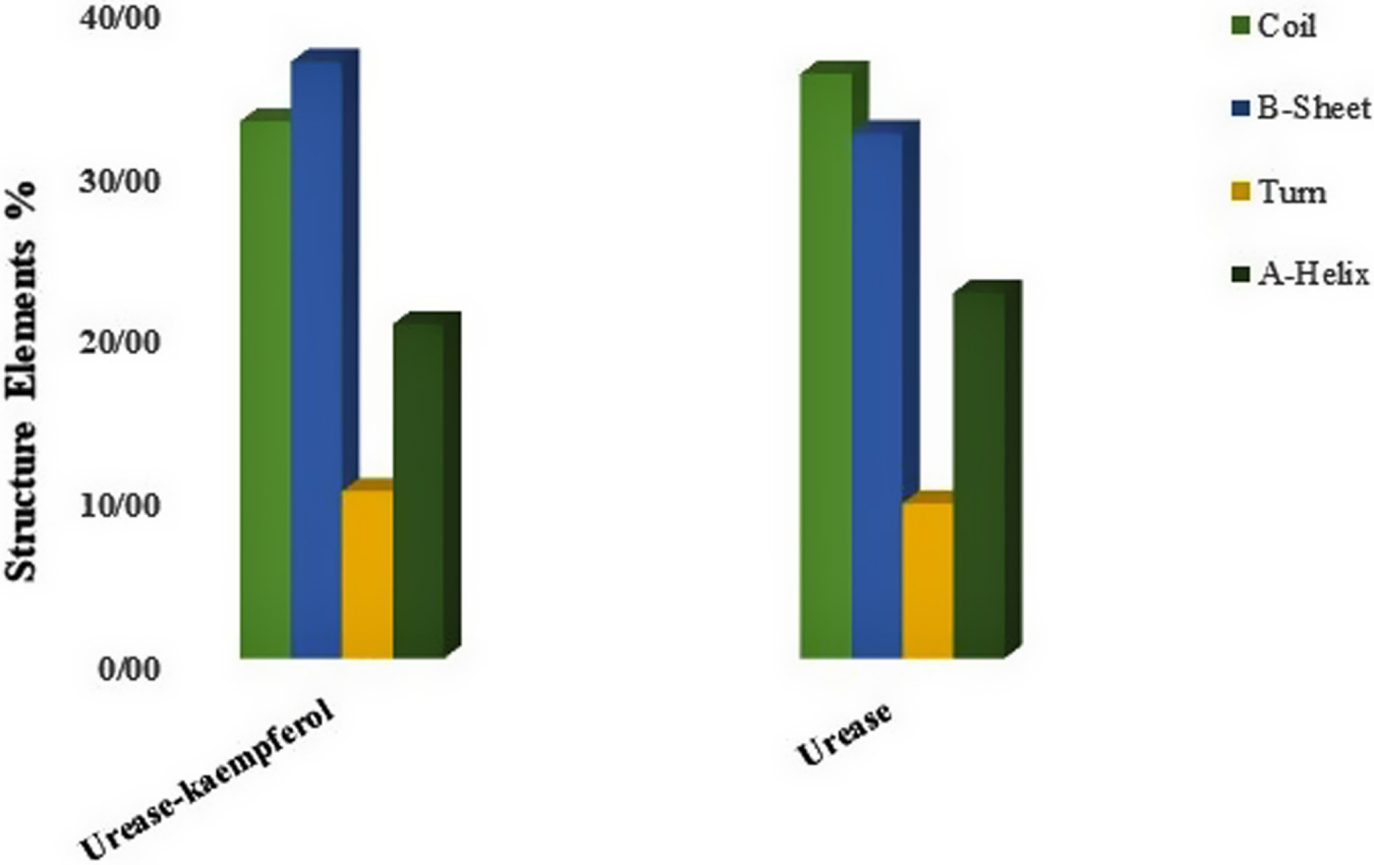
The secondary structure (DSSP algorithm) for urease–kaempferol and urease

Another important feature of GROMACS software is secondary structure analysis of proteins using the algorithm DSSP. The content of α‐helix decreased in the presence of an inhibitor, indicating further instability of the system (Figure 7). The reduction in α‐helix (22.36 to 20.44), which is a significant component of the secondary structure of proteins, is clear evidence of the instability and disruption of the urease structure in the presence of kaempferol (Pretzler et al., 2017).

In addition, binding free energy of kaempferol was measured by applying MM‐PBSA calculations (Massova & Kollman, 2000). The binding free energy consists of polar solvation, Van der Waals interactions, electrostatic, and SASA energy. Contribution of electrostatic, Van der Waals, and SASA energy were negative, whereas positive contributions were shown by polar solvation energy to the overall free binding energy. As shown in Table 3, Van der Waals energy was observed to be the primary benefactor for the interaction of kaempferol with urease. These results are also in accordance with the effect of kaempferol on protein‐15 of SARS‐CoV‐2 (Sharma et al., 2021). The contribution of each residue was also explored in Figure 2s, where the higher the contribution of a residue toward favorable interaction, more negative is energy value, whereas unfavorable contribution attains a positive energy value. High contributing residues of urease are Kcx, −0.08923 kJ/mol, His519, −1.4258 kJ/mol, His545, −4.0231 kJ/mol, Gly550, −2.03 kJ/mol, Cme592, −4.852 kJ/mol, and Asp633, −0.95863 kJ/mol, with two weak contributing residues Ala636 and Met637.

**TABLE 3:** MMPBSA‐based total binding free energies along with its constituent energies for urease–kaempferol

Complex	Total binding free energy (KJ/mol)	Van der Walls energy (KJ/mol)	Electrostatic energy (KJ/mol)	Polar salvation energy (KJ/mol)	SASA energy (KJ/mol)
Urease–kaempferol	−51.578	−97.686	−21.321	79.232	−11.009

### 2.10 Comparison of molecular dynamics with **extended solvation model**

In general, the results of molecular dynamics and kinetics confirm the inhibition of the urease enzyme by kaempferol. It is fascinating that these results have been developed following the thermodynamic study of inhibition by the extended solvation model. As shown in Tables 1 and 2, the interaction is entropy driven, indicating that the vdW energy is dominant, and docking and MMPBSA confirm this. Negative δ_A_ and δ_B_ values indicate the formation of an unstable complex of urease with kaempferol. In parallel, the results of molecular dynamics and increasing *R*_g_ value and hydrophobic amino acids accessible in the solvent confirmed the system's unfolding in the presence of kaempferol. On the other hand, close changes in the amount of δ_A_ and δ_B_ characterize particular space interactions in the system. P > 1 and negative δA and δB show that kaempferol causes a few reversible changes in the urease structure. Affinity binding kaempferol to urease, reduction in activity urease, and binding confirmations from molecular simulation studies also confirm the noncompetitive inhibition type, as shown in Figure 2. Meanwhile, the total binding free energy obtained from ITC and extended solvation model was almost close to the MMPBSA result, as shown in Table 2 and 3. Finally, comparing the results of MD with the previous study, it can be seen that the solubility extended solvation model (Behbehani et al., 2009; Behbehani et al., 2016; Behbehani & Barzegar, 2013; Poursoleiman et al., 2019; Rezaei Behbehani, Saboury, Mohebbian, et al., 2010). On many enzymes and inhibitors used in vitro is a successful model that, along with molecular dynamics and simulation, can help researchers in medicine and enzymes and physicians and patients.

## 3 CONCLUSION

This study reports the molecular dynamics of urease–kaempferol complexes, molecular docking, MM‐PBSA, and urease inhibitory activities. The molecular docking and molecular dynamics studies on the kaempferol versus Jack bean urease valuably led to the development of a new urease inhibitor. This molecule formed interactions with key residue His and it formed H‐bonds and van der Waal interactions with other residues, which shows that the selected molecule has significant binding interactions within the active site residues. The inhibitory activity tested in vitro against Jack bean urease shows that kaempferol exhibits a partially good inhibitory activity of IC50, 6.96 μM. Importantly, we only focused on comparing and confirming the results; detailed research continuously examined the toxicity of these complexes of urease inhibitory activity posed to the environment and humans.

## CONFLICT OF INTEREST

The authors declare that they have no competing interests.

## Supporting information


Figure S1

Figure S2

Table S1

## Data Availability

The datasets used and/or analysed during the current study are available from the corresponding author on reasonable request.
