# Supplementary figures and images for: Molecular dynamics simulations, molecular docking, and kinetics study of kaempferol interaction on Jack bean urease: Comparison of extended solvation model
Source: Food Sci Nutr. 2022 Jul 2;10(11):3585–97. doi: 10.1002/fsn3.2956 (PMC9632207; doi:10.1002/fsn3.2956)

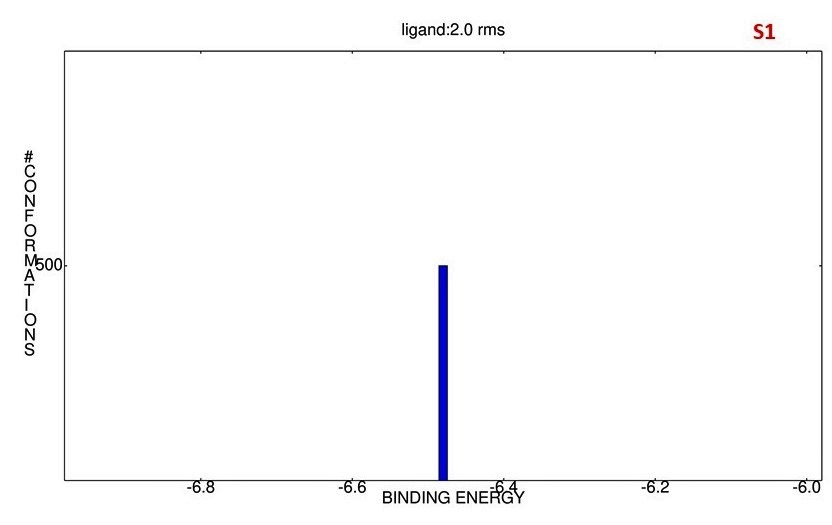

Supplement: Supplementary file 1 — Figure S1 [file FSN3-10-3585-s001.jpg]

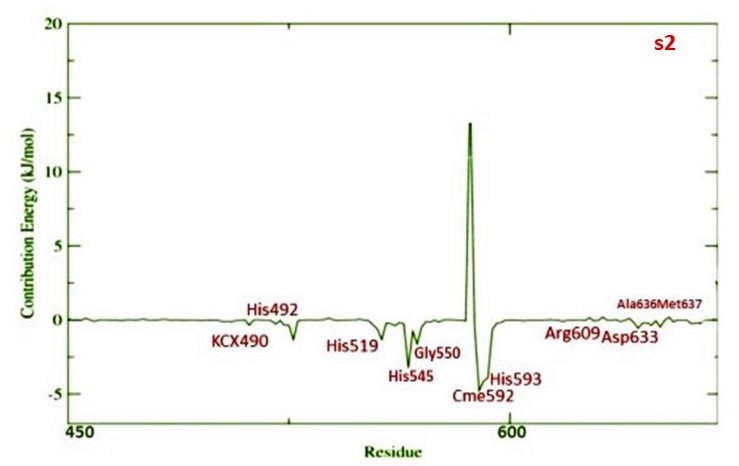

Supplement: Supplementary file 2 — Figure S2 [file FSN3-10-3585-s003.jpg]
