# Supplementary material for: Molecular dynamics simulations, molecular docking, and kinetics study of kaempferol interaction on Jack bean urease: Comparison of extended solvation model
Source: Food Sci Nutr. 2022 Jul 2;10(11):3585–97. doi: 10.1002/fsn3.2956 (PMC9632207; doi:10.1002/fsn3.2956)
Supplement: Supplementary file 3 — Table S1 [file FSN3-10-3585-s002.docx]

**Supplementary Table 1: The total binding energies obtained after docking**

|  | NO. of strucures | Binding Energy (Kcal/mol) | Ligand Efficiency | Ki(µM) | Intermol Energy (Kcal/mol) | VdW Energy (Kcal/mol) | Elec. Energy (Kcal/mol) |
| --- | --- | --- | --- | --- | --- | --- | --- |
| Cluster1 | 500 | -6.48 | -0.31 | 17.92 | -7.97 | -7.79 | 0.17 |
